# Mixed-Potential Ammonia Sensor Based on a Dense Yttria-Stabilized Zirconia Film Manufactured at Room Temperature by Powder Aerosol Deposition

**DOI:** 10.3390/s24030811

**Published:** 2024-01-26

**Authors:** Nils Donker, Daniela Schönauer-Kamin, Ralf Moos

**Affiliations:** Department of Functional Materials, University of Bayreuth, Universitätsstraße 30, 95440 Bayreuth, Germany

**Keywords:** NH_3_ sensor, powder aerosol deposition (PAD), YSZ, mixed potential, SCR catalyst

## Abstract

Powder aerosol deposition (often abbreviated as PAD, PADM, or ADM) is a coating method used to obtain dense ceramic films at room temperature. The suitability of this method to obtain ammonia mixed-potential sensors based on an yttria-stabilized zirconia (YSZ) electrolyte that is manufactured using PAD and a V_2_O_5_–WO_3_–TiO_2_ (VWT)-covered electrode is investigated in this study. The sensor characteristics are compared with data from sensors with screen-printed YSZ solid electrolytes. The PAD sensors outperform those in terms of sensitivity with 117 mV/decade NH_3_ compared to 88 mV/decade. A variation in the sensor temperature shows that the NH_3_ sensitivity strongly depends on the sensor temperature and decreases with higher sensor temperature. Above 560 °C, the characteristic curve shifts from exponential to linear dependency. Variations in the water and the oxygen content in the base gas (usually 10% oxygen, 2% water vapor in nitrogen) reveal a strong dependence of the characteristic curve on the oxygen content. Water vapor concentration variations barely affect the sensor signal.

## 1. Introduction

Ammonia (NH_3_) has long been known as an essential component of fertilizers. The Haber–Bosch process allows for the production of ammonia on an industrial scale. Today, an estimated 80% of the ammonia produced via the Haber–Bosch process is used in the manufacture of fertilizers [1]. In addition, NH_3_ is also used as a refrigerant and for exhaust gas aftertreatment. In the latter, NH_3_ is injected into the exhaust gas stream in the form of an aqueous urea solution. In a selective catalytic reduction (SCR) catalyst, nitrogen oxides (NO_x_) are reduced to harmless nitrogen (N_2_) and water (H_2_O) [2]. Such systems are used in exhaust gas aftertreatment of power plants, diesel car engines, or—more generally—for lean-operation combustion processes. Examples are given in [3,4].

In addition to these already widespread applications, global climate change and the resulting conversion to renewable energy sources are bringing even more fields of application for NH_3_. Most renewable energy sources are not reliably available at all times. This makes energy storage necessary, for instance, in the form of chemical substances such as hydrogen (H_2_). H_2_ as a carbon-free species can easily be produced from surplus electrical energy and water through electrolysis [5]. H_2_ can be re-used as fuel or processed into NH_3_. NH_3_ offers significant advantages over hydrogen because it is easier to store and transport [6]. On the one hand, there are safety issues because hydrogen is volatile, has a low flash point, is explosive in air, and has an invisible flame. On the other hand, hydrogen has a low volumetric energy density, even in the liquid state at −253 °C. The cost of NH_3_ per unit of stored energy volume is approximately three times lower than that of hydrogen [7]. NH_3_ can be transported and used directly as a fertilizer or as a component for NH_3_ fuel cells. Additionally, H_2_ can be regained from NH_3_ [8].

Sensitive and selective NH_3_ sensor systems are, therefore, necessary for many different fields. Applications are leakage detection to prevent dangerous situations, as well as monitoring of air quality and preventing overdosing in exhaust gas aftertreatment SCR systems.

Research on various gas sensor types, including metal oxide gas sensors, NH_3_ sensors made of conductive polymers, and optical NH_3_ detection methods, has been published (see overview in [9]). Metal oxide gas sensors have the advantage of being simple and inexpensive. However, as with many gas sensors, selectivity is a problem, especially with mixtures of several gas components. Ammonia sensors made of conductive polymers offer advantages such as ease of manufacture and modification, stability, flexibility in design, and compatibility with other materials. The comparatively long recovery times are a disadvantage here. They are also more suitable for applications at low temperatures; for applications in exhausts, they are not stable enough. Optical ammonia sensors show good selectivity but require an expensive setup. However, the first two types have low selectivity, the conductive polymers are not reversible, and the optical methods require an expensive setup [9]. Some electrochemical sensors follow the mixed-potential principle. They are also denoted as non-equilibrium sensors. They are often based on yttria-stabilized zirconia (YSZ) as the oxygen-ion-conducting solid electrolyte [10,11,12,13,14,15,16]. In addition, various metal oxides are used directly as sensor electrodes [17,18,19,20,21] or on top of a noble metal electrode [22,23,24,25,26,27,28]. A second noble metal electrode such as gold or platinum acts as a reference. Sensors of this type are already commercially available [29,30].

At these electrodes, two or more electrochemical reactions compete. In case of NH_3_ mixed-potential sensors, the dominating electrochemical reactions are [23]

(1) $$\frac{1}{2}O_{2}+2e^{\prime }+V_{O}^{••}⇌O_{o}^{x}$$

(2) $$\frac{2}{3}NH_{3}+O_{o}^{x}⇌H_{2}O+\frac{1}{3}N_{2}+2e^{\prime }+V_{O}^{••}$$

where $V_{O}^{••}$ denotes a double positively charged oxygen vacancy, $O_{O}^{x}$ denotes an oxide ion on an oxygen site according to the Kröger–Vink defect notation [31], and e′ stands for a conduction electron. 

When only one electrochemical reaction takes place at an electrode, an equilibrium potential is formed at the electrode according to the Nernst equation. In this case, the anodic and cathodic reactions take place at the same rate. Both the net current and the conversion rate are, therefore, zero. In the case of a mixed potential, several individual reactions take place simultaneously at this electrode. Again, a potential is established at which the net current is zero. However, this net current is considered over all the individual reactions. The higher the individual current of a single reaction, the more it contributes to the mixed potential. A more detailed explanation can be found in [10]. To measure a potential difference between the electrodes, which is the sensor signal, one of the reactions must be promoted at one of the electrodes. Therefore, the electrodes are either exposed to different atmospheres, different electrode materials are used [32], or the thermodynamic conditions (“atmosphere”) at one of the electrodes are changed, for example, by an additional catalytic layer at this electrode [10]. Many published ammonia mixed-potential sensors use SCR active materials for that purpose, such as vanadium oxide or tungsten oxide [21,29,30,33,34,35,36]. A mixture of these materials, V_2_O_5_–WO_3_–TiO_2_ (VWT), is often used as such a catalytic layer [23,25,26,27,28,37]. VWT is a commercially available material that is well-known as a harsh-environment stable catalyst for SCR exhaust gas aftertreatment applications. Various properties have already been investigated for this type of sensor, such as variations in the vanadium content [26,27] or its sintering temperature [28]. In addition, the half-cell potentials were examined using a two-chamber design [25]. 

In order to heat the (mostly planar) sensors, a heater is required. It must be electrically insulated from the sensor. For planar sensors, the individual layers are typically applied to an alumina substrate via screen printing [23,37] or e-beam evaporation [19]. A new manufacturing process for such dense ceramic layers is the powder aerosol deposition method (often abbreviated as PAD, PADM, or ADM). It is a coating method to obtain dense ceramic films at room temperature [38,39,40,41,42]. Compared to screen-printed thick-films, which are usually not dense, it is reported that they perform better with respect to their ionic or electronic conductivity [43]. Furthermore, the film adhesion is reported to be excellent [44]. Since YSZ already adheres at room temperature to the substrate, no additional binders, as are required for screen-printing of YSZ films, are needed. And since no additional sintering step occurs, interdiffusion from aluminum to YSZ or from yttria or zirconia does not occur.

PAD can be used to deposit not only protective or insulating layers such as alumina [45] but also other functional materials such as ion conductors [46,47,48,49,50,51,52] or thermoelectric materials [53,54]. The suitability of such layers for sensor applications, also with YSZ as solid electrolyte, has already been demonstrated [55,56].

This study aimed to investigate whether NH_3_ mixed-potential gas sensors based on PAD-YSZ electrolyte layers and VWT as the electrode cover can be manufactured and how they perform compared to their screen-printed counterpart. Note that the vanadium paste used for the catalytic layer in this study was identical to that used in [23].

## 2. Experimental

### 2.1. Sensor Setup

**Figure 1:**
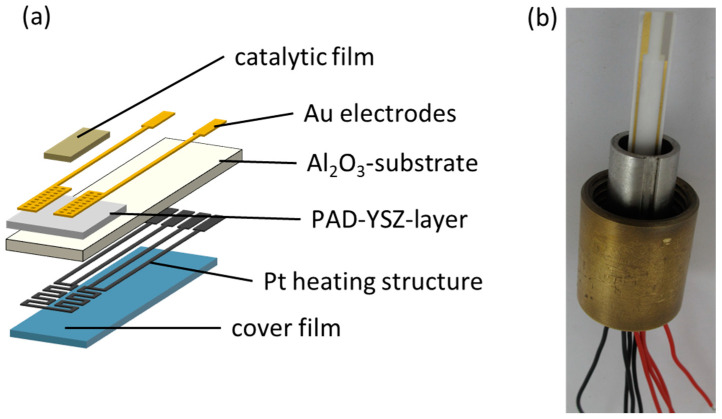
Structure of the mixed-potential ammonia sensor with PAD solid electrolyte layer as (**a**) schematic view and (**b**) sensor photo.

The tested NH_3_ sensors were formed using a ceramic structure with multiple layers (Figure 1), including an insulating alumina substrate, an YSZ-based solid electrolyte (PAD), and gold electrodes, of which one was catalytically coated with a porous VWT film. The electrolyte was deposited directly onto the substrate using PAD, which will be described later in the text. A platinum heating structure was printed on the reverse side of the alumina substrate and fired at 1150 °C. The substrate acted as electrical insulation between the heating side and the sensor side. A cover layer was also screen-printed on top of the heating structure and fired at 850 °C to protect the heater and to prevent gas reactions (especially ammonia oxidation) at the hot platinum layer, to ensure that the full NH_3_ concentration reached the sensor electrodes where the signal was formed [57,58]. The gold electrodes were screen-printed on top of the PAD-YSZ solid electrolyte layer and fired at 850 °C. The later-added porous VWT cover, which was identical to that used in [23], was fired at 600 °C. The VWT was chosen because it was a porous, commercially available SCR catalyst with proven long-term stability in the exhaust. Since the VWT had already achieved good results and the paste was already available, it was used for comparison. The purpose of using a VWT catalyst film is to change the thermodynamic conditions at an electrode. In this way, eight sensors are produced at the same time. The sensors have similar characteristics, with only minor differences.

The sensor temperature was controlled using an external heater control circuit. The temperature-dependent resistance of the platinum heater (measured in four-wire configuration) was used as the controlled variable. The heating power was adjusted so that the heater resistance corresponded to the setpoint temperature. Through that approach, the resistance of the heater was set to around 0.01 Ohm. Converted to a temperature, this corresponded to approx. 0.4 °C.

The signal of the sensor was measured as a voltage between both electrodes. Voltages were recorded every second as open circuit voltage (OCV) by using a measuring device for electrochemical analysis (PalmSens4, PalmSens BV, Houten, The Netherlands) with a resolution of 7.8 µV.

### 2.2. Powder Aerosol Deposition Method

The solid electrolyte layer was prepared using the powder aerosol deposition method (PAD). Firstly, 8YSZ powder (TZ-8YS, TOSOH Corporation, Tokyo, Japan) was calcined at 1250 °C for 10 h in air, followed by milling in cyclohexane for 3 h, then dried and sieved through a 90 µm sieve to break up larger agglomerates. The resulting powder was stored within a 200 °C drying oven for several days to remove residual moisture.

**Figure 2:**
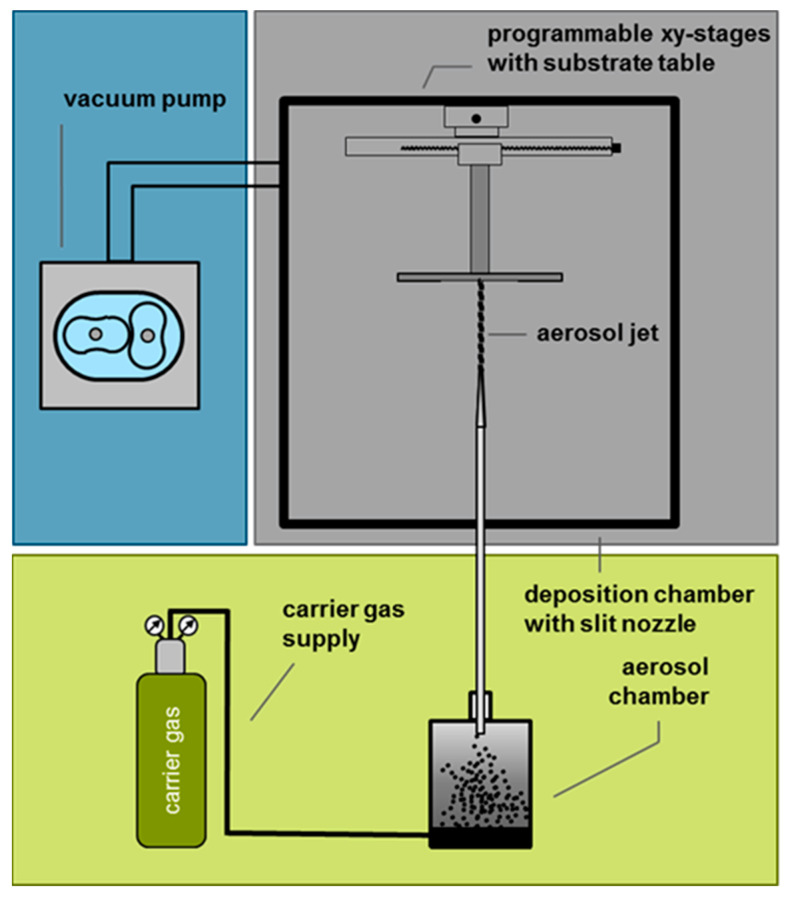
Schematic of a PAD apparatus.

**Figure 3:**
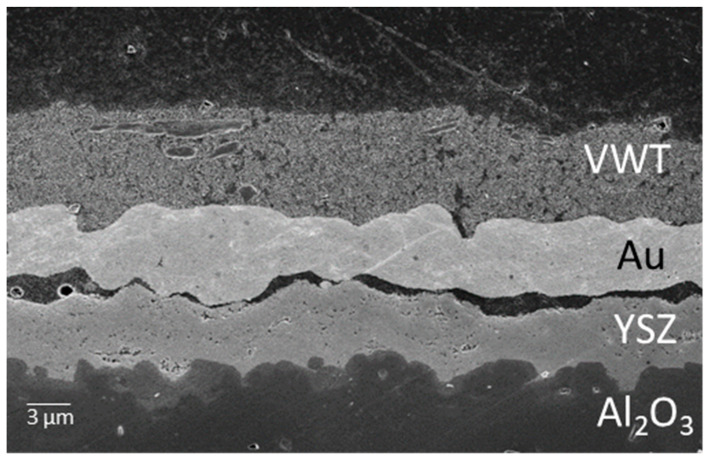
Scanning electron microscope image of a PAD solid electrolyte layer with a gold electrode and a catalytic VWT layer on top. The distance between the layers of Au and YSZ is due to the sample preparation, as a conductive connection was required for a sensor signal; the gap cannot, therefore, have occurred during operation. The YSZ-Al_2_O_3_ interface is very dense and YSZ adheres very well to the Al_2_O_3_ substrate.

The PAD apparatus for coating the planar devices is outlined in Figure 2. Here, the substrate is placed in a vacuum chamber on an x-y moveable table. A powder aerosol is generated as described in detail in the literature [46]. O_2_ serves as a carrier gas. The powder aerosol is accelerated by the pressure difference through a slit nozzle. The YSZ particles then impact the substrate, break up, and form a dense, adherent layer with nanocrystalline morphology (Figure 3). The size of this layer can be adjusted by moving the substrate table and the layer thickness by the duration of the deposition process. Regarding the phase of the deposited layer, it has been shown that the original phase of the starting material can be retained in the deposited layer. For further details on PAD-YSZ layers, such as the crystal structure or conductivity, please see [42,46].

### 2.3. Measurements

**Figure 4:**
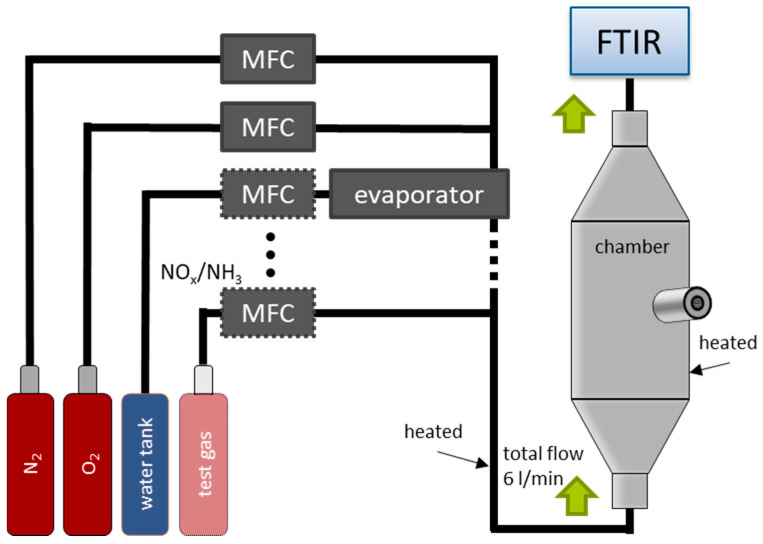
Schematic view of the sensor test chamber with the respective test gases, the chamber, and the FTIR for analysis.

The sensor measurements were conducted in a gas mixing system, shown schematically in Figure 4. The respective test gases (NO, NO_2_, NH_3_) and base gases (N_2_, O_2_) are admixed via mass flow controllers (MFCs) at a total flow rate of 6000 mL/min. In addition, water (H_2_O) is evaporated, mixed with the gases, and fed into the gas stream. The base gas consists of 10% O_2_, and 2% H_2_O and N_2_ as a balance. During the measurements, NO, NO_2_, and NH_3_ were added pulse-wise to the base gas for 10 min each. Both the gas supply lines and the test chamber were heated to approx. 200 °C to prevent adsorption of NH_3_ or NO_2_. The sensors (Figure 1b) were screwed into the measuring chamber and operated. Downstream of the chamber, a Fourier-transform infrared spectroscope (FTIR) (MKS MultiGas 2030 FTIR Analyzer, MKS instruments, Andover, MA, USA) analyzed the actual gas composition. These measured concentrations were used as references.

## 3. Results and Discussion

### 3.1. Sensor Temperature Dependence

**Figure 5:**
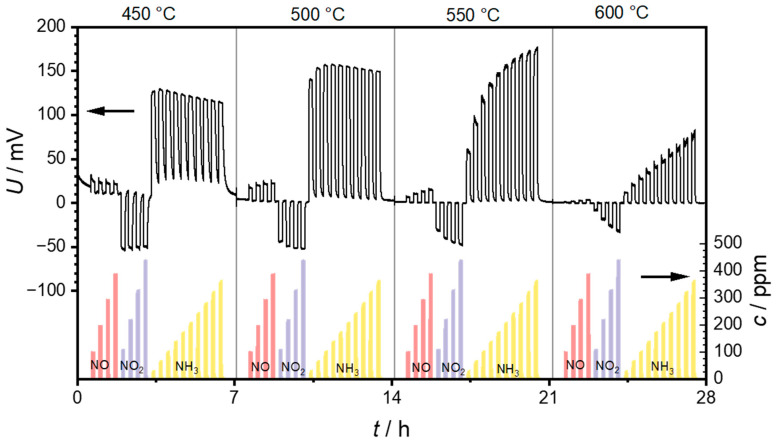
Measurement of the sensor at operating temperatures of 450, 500, 550, and 600 °C, with repeated exposure to NO, NO_2_, and NH_3_. The left axis shows the sensor voltages measured between the two electrodes, and the right axis shows the gas concentrations of NO, NO_2_, and NH_3_ measured with the FTIR. Each temperature step lasted for 7 h.

The first set of measurements was intended to test the general suitability of the PAD-YSZ layer. For this purpose, as well as to determine the optimum operation temperature, the sensor temperature was changed stepwise in 50 °C increments from 300 to 600 °C. Each of these temperature steps lasted 7 h. At each temperature, NO, NO_2_ and finally NH_3_ were added with an increasing concentration. Figure 5 presents the recorded voltages of a sensor and the concentrations of NO, NO_2_, and NH_3_ as measured via FTIR. At temperatures below 450 °C, the voltages were too noisy, which is why they are not shown here.

It can be seen that positive voltage changes can be measured when NO is added to the base gas. These voltage changes decrease with increasing operation temperature and can hardly be identified at a sensor temperature of 600 °C. In contrast, the voltage changes induced by NO_2_ are negative. They also decrease with increasing sensor temperature. However, the concentration dependence of the voltage changes increases with increasing sensor temperature. A saturation effect can be observed at 450 and 500 °C while exposed to NO_2_, which means that the voltage difference is independent of the NO_2_ concentration. This saturation can no longer be observed at sensor temperatures above 550 °C, and a clear dependence of the sensor voltage on the NO_2_ concentration is recognizable. Similarly to NO, NH_3_ also causes a positive voltage change. Here, the signal amplitudes increase from 450 to 500 °C. However, there is no clear dependence on ammonia concentration. The voltages even tend to decrease with increasing ammonia concentration. This changes at 550 °C, where the voltage changes increase with increasing ammonia concentration. At 600 °C, this concentration dependence is still present, but the voltages are significantly lower compared to 550 °C.

Unfortunately, due to the time delays caused by the gas path and the resulting non-rectangular concentration profile, it is not possible to define an exact response behavior such as the *t*_90_ time. However, it can generally be said that the response behavior is in the range of a few seconds. It is also not possible to specify an exact time for the recovery time of the sensor. Therefore, it can be observed that the response becomes faster as the sensor temperature increases. This may be due to the faster kinetics on the one hand, but also to the lower sensitivity at higher temperatures on the other.

**Figure 6:**
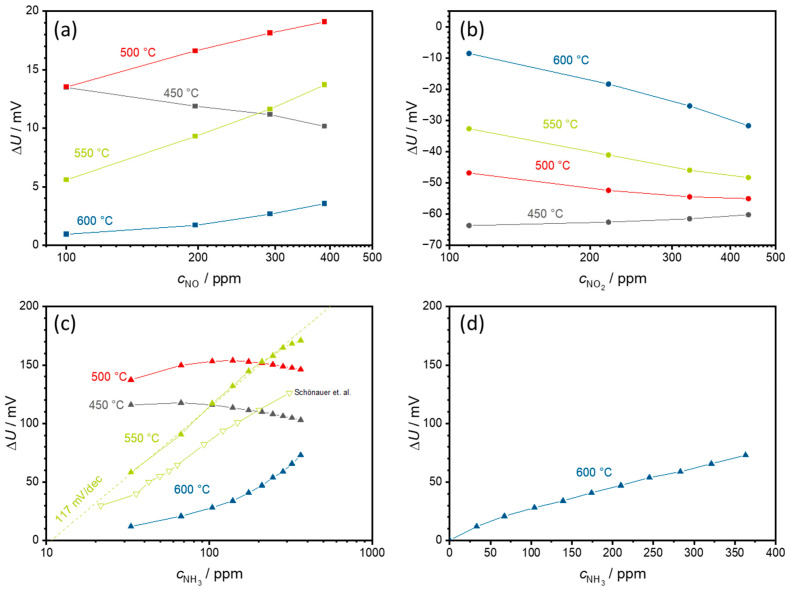
Semilogarithmic characteristic curves of the measurement shown in Figure 5 for (**a**) NO, (**b**) NO_2_, and (**c**) NH_3_ in semilogarithmic scale, and (**d**) NH_3_ in linear scale. In addition, (**c**) includes a comparison with measurements from a screen-printed solid electrolyte and with an otherwise identical structure taken from [23]. (**d**) illustrates the linear NH_3_ dependence at a sensor temperature of 600 °C, which was semilogarithmic at lower temperatures. Please note the different scaling of the axes. The points correspond to the sensor voltages measured at each concentration level. The lines that connect these points serve only as a guide for the eye.

Figure 6 was obtained from the data shown in Figure 5 by calculating the voltage difference ∆*U* between the measured voltage in the respective test gas and the measured voltage in the base gas and plotting these voltage differences in relation to the test gas concentration. The slope of this characteristic curve is the sensitivity (in mV per decade due to the exponential dependency).

Figure 6a shows these characteristic curves for NO in a semilogarithmic representation. At 450 °C, ∆*U* decreases with increasing NO concentration. At 500 °C and above, there is a dependency of the sensor signal on the NO concentration. However, the signal remains below 20 mV for all cases. 

Figure 6b illustrates the sensor characteristics for NO_2_. A negative voltage difference ∆*U* is measured here. The largest magnitude of the signal amplitude occurs at 450 °C with −63 mV, but with almost no NO_2_ concentration dependence. As the sensor temperature increases, the absolute ∆*U* decreases, but the concentration dependence, i.e., the slope, of the NO_2_ signal increases. This leads to the observation of a semilogarithmic characteristic curve with a maximum slope of −38 mV/decade NO_2_ at 600 °C. However, the maximum ∆*U* of −32 mV at 437 ppm NO_2_ is also the lowest at 600 °C.

The highest signal amplitudes ∆*U* up to 150 mV at 500 °C were obtained with NH_3_, as shown in Figure 6c. The concentration dependence at 450 and 500 °C is not clear. Hence, ∆*U* first increases and then decreases again at higher ammonia concentrations. Significant changes in the sensor behavior are observed at 550 °C. Here, a semilogarithmic concentration dependence of ∆*U* with a sensitivity of about 117 mV/decade NH_3_ is observed. When comparing the sensors formed via PAD with screen-printed sensors, not only are the signal amplitudes significantly higher at this temperature but also the slope of the characteristic curve is higher, with 117 mV/dec NH_3_ compared to the 88 mV/dec presented in [23]. This demonstrates the good suitability of the PAD solid electrolytes for these sensors. At 600 °C, the NH_3_ behavior changes to a linear dependence with a slope of 0.18 mV/ppm NH_3_ but with a clearly smaller signal change ∆*U*. The linear dependence is shown in Figure 6d.

**Figure 7:**
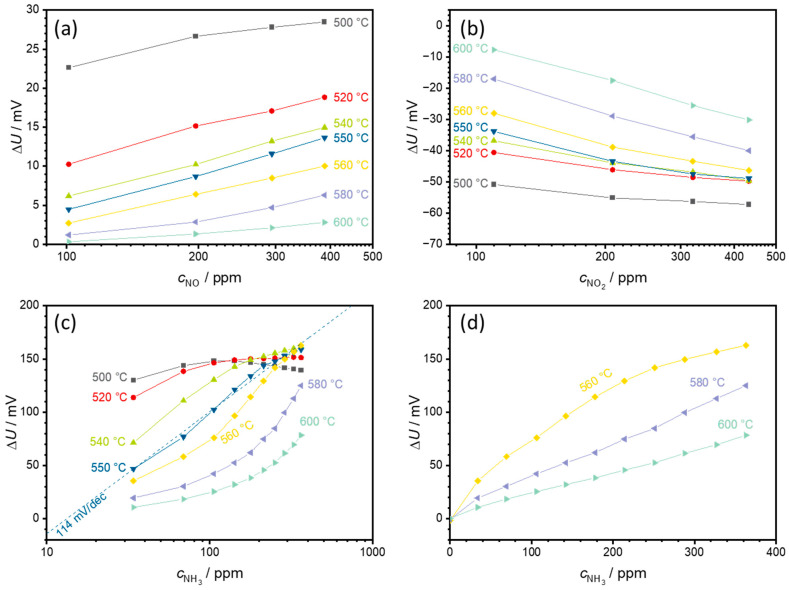
Semilogarithmic characteristic curves of a sensor for sensor temperatures from 500 to 600 °C for (**a**) NO, (**b**) NO_2_, and (**c**) NH_3_ as well as (**d**) the linear NH_3_ dependency at higher temperatures. (**c**,**d**) show the transition from a semilogarithmic to a linear ammonia characteristic curve in the temperature range between 560 and 580 °C, i.e., the sensor behavior changes from exponential to linear behavior. The points correspond to the sensor voltages measured at each concentration level. The lines that connect these points serve only as a guide for the eye.

To obtain a more accurate insight into the sensor behavior at different temperatures, additional test runs were performed in a smaller temperature range between 500 and 600 °C in steps of 20 °C. In addition, the sensor was measured again at 550 °C to exclude possible aging effects during the measurement. The resulting semilogarithmic characteristic curves for NO, NO_2_, and NH_3_ are shown in Figure 7.

For all gases investigated (NO, NO_2_, and NH_3_), the sensor voltage differences ∆*U* decrease with increasing temperature. As can be seen in Figure 7a, there is only a small concentration-dependent NO signal with a maximum ∆*U* of 28.5 mV at a NO concentration of 388 ppm and at a sensor temperature of 500 °C. As the sensor temperature increases, this drops further to 2.8 mV at 600 °C.

In the case of NO_2_ (Figure 7b), a small concentration dependence appears at 500 °C. With increasing temperature, the absolute measured ∆*U* decreases, whereby the slope of the characteristic curve increases up to −38 mV/decade NO_2_ at 600 °C. The behavior is comparable to the measurement shown in Figure 6.

The response to NH_3_ is shown in Figure 7c. Compared to NO or NO_2_, ∆*U* is significantly higher. At a sensor temperature of 500 °C, ∆*U* is highest, but as with the measurement shown in Figure 6, this is not clear. Two effects occur with increasing sensor temperature: the voltage difference ∆*U* decreases, and the concentration dependence of the characteristic curve increases. At 550 °C, a slope of around 114 mV /dec NH_3_ can be observed. This is very close to the previous measurement at 550 °C. Above this 550 °C, the characteristic curve of the sensor characteristics changes from exponential to linear behavior (from linear behavior in a semilogarithmic representation to a constant slope in a linear representation), as shown in Figure 7d. At 580 °C, a clear linear dependence is observed with a slope of 0.33 mV/ppm NH_3_. This decreases to 0.20 mV/ppm NH_3_ at 600 °C.

As this is an ammonia sensor, the temperature-dependent behavior is explained using reactions (1) and (2) for the ammonia signal. Similar arguments apply to NO and NO_2_. For the corresponding electrochemical reactions, see [10].

This temperature-dependent sensor behavior might be explained by several individual mechanisms. The noisy signals at temperatures below 450 °C could be explained by the high electrolyte resistance and the slower kinetics of the electrochemical reactions (1) and (2). As a result of these, even the smallest currents influence the sensor behavior during voltage measurement, which makes it difficult to obtain stable signals. Above a sensor temperature of 450 °C, these noise effects appear to be negligible.

If only reactions (1) and (2) are considered, the mixed potential is the potential at which both reactions proceed at the same rate [32]. This mixed potential is located between the equilibrium potentials of the individual reactions. The more dominant one of these reactions is, the closer the mixed potential is to the equilibrium potential of this reaction. At 450 °C and 500 °C, the ammonia reaction (2) seems to be so dominant that the sensor shows saturation This means that more ammonia would hardly accelerate this reaction (2), which is why ∆*U* is almost independent of the ammonia concentration. Lower ammonia concentrations in the gas could weaken this reaction (2), which is why it might be possible to detect lower ammonia concentrations at these sensor temperatures.

With increasing temperatures above 500 °C, the independence of the ammonia concentration vanishes increasingly, indicating that the oxygen reaction (1) has a greater effect on the potential compared to the ammonia reaction (2). This may be due both to accelerated oxygen kinetics at higher temperatures [59] and to faster desorption of the ammonia [28,60]. 

An explanation for the linear characteristic at high sensor temperatures is given by Garzon et al. [61]. Here, a case is discussed in which the reaction is not limited by the reaction kinetics but by mass transport. However, transport limitation alone will still lead to a logarithmic dependence on concentration and electrode potential. The same is valid if the mixed potential is close to the equilibrium of the oxygen reaction. However, if both cases occur simultaneously, the mixed potential is close to the equilibrium potential of the oxygen reaction, and the ammonia reaction is transport limited, a linear relationship between electrode potential and ammonia concentration will result. This explains the linear relationship observed at sensor temperatures above 560 °C.

**Figure 8:**
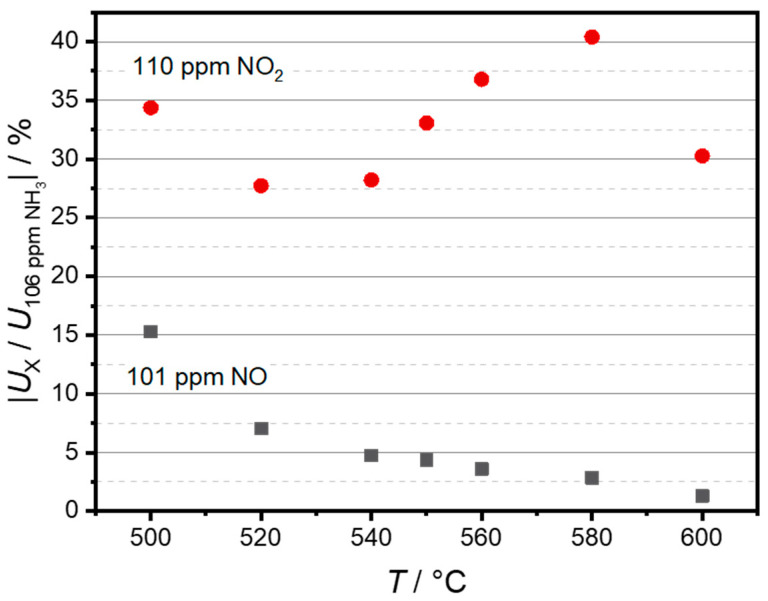
Sensor selectivity is indicated by the signal change of 101 ppm NO or 110 ppm NO_2_ relative to the signal change of 106 ppm NH_3_.

To further visualize the temperature dependence of the NO and NO_2_ cross-sensitivities, Figure 8 shows a comparison between sensor voltages at approximately 100 ppm of NO and NO_2_ (*U*_x_) and the voltage at 100 ppm of NH_3_ *U*_NH3_ in %. Please note that *U*_x_/*U*_NH3_ is presented as an absolute value. The highest NO cross-sensitivity can be observed at 500 °C, accounting for 15.3% of the NH_3_ sensitivity. The sensitivity decreases with increasing temperature and has its minimum at 600 °C with 1.3%. As both NO_2_ and NH_3_ sensitivity also decline with the increase in temperature, there is no apparent correlation between selectivity and sensor temperature. In the investigated temperature range, the relative NO_2_ cross-sensitivity was lowest at 520 °C with 27.7%. The highest relative NO_2_ cross-sensitivity, which was 40.4%, was observed at 580 °C. Due to the inverted polarity, though, these gases can be simply distinguished in sensor measurements as long as they appear separately.

The sensor was operated for several days during the temperature variations. Complete long-term stability was not investigated (however, it should be noted that the sensors were operated for 70 h at a minimum of 500 °C). This is planned for the future.

### 3.2. Effects of O_2_

Since the oxygen exchange reaction (1) plays an important role in sensor behavior, the influence of other oxygen concentrations in the base gas was also investigated. For this purpose, the sensor temperature was kept constant at 550 °C.

**Figure 9:**
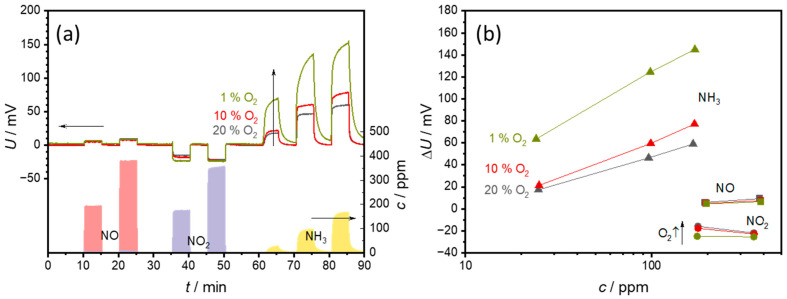
Effect of an oxygen concentration of 1%, 5%, or 10% oxygen in the base gas (2% H_2_O in N_2_) on (**a**) the sensor signal and (**b**) the sensor characteristic curves. The points correspond to the sensor voltages measured at each concentration level. The lines that connect these points serve only as a guide for the eye.

Figure 9 illustrates the sensor voltage (Figure 9a) and the characteristic sensor curves (Figure 9b) in the base gas containing 1%, 10%, or 20% oxygen and constant 2% H_2_O in N_2_. 

As can be seen in Figure 9a, the baseline without test gases (NO, NO_2_, NH_3_) remains largely unaffected by the changes in oxygen content. The response of the sensor to NO and NO_2_ also remains largely unaffected by changes in the oxygen concentration. In contrast, the sensor response to NH_3_ is significantly affected by changes in oxygen concentration. A lower oxygen concentration leads to a stronger reaction of the sensor to NH_3_, and also to a significantly slower reaction time. Thus, no stable voltage was achieved at 1% O_2_ during the 5 min NH_3_ pulse.

The characteristic curves in Figure 9b illustrate the same situation. In the case of NO, barely any dependence on the oxygen concentration can be seen. For NO_2_, an oxygen dependence can be recognized at 175 ppm NO_2_. Here −24 mV is measured at 1% O_2_, compared to −16 mV at 20% O_2_. These differences become less pronounced as the NO_2_ content increases. The oxygen influence is strongest together with NH_3_. Here, the ∆*U* of 144 mV at 170 ppm NH_3_ and 1% O_2_ is almost three times as high as that for 59 mV at 20% O_2_. The sensitivity, i.e., the slope of the characteristic curve, also increases with decreasing oxygen content.

The strong oxygen dependence is to be expected due to the sensor principle. With a lower oxygen content, the ammonia reaction (2) becomes dominant rather than the oxygen reaction (1). As a result, the ammonia contributes more to the sensor voltage. The slower sensor response indicates slower reaction kinetics. As a result, it would take longer for a stable electrode potential and thus a stable state to be established. As this occurs at lower oxygen contents, a slower reaction (1) is probably the reason for this. The slower establishment of a stable signal can affect one or both electrodes. Schönauer-Kamin et al. have shown that the electrode covered with VWT is primarily responsible for the ammonia signal [25], but for a stable signal, both potentials must be stabilized.

Nevertheless, the strong dependence on oxygen makes it necessary to determine the oxygen content for sensor applications in order to be able to compensate the effects.

### 3.3. Effects of H_2_O

**Figure 10:**
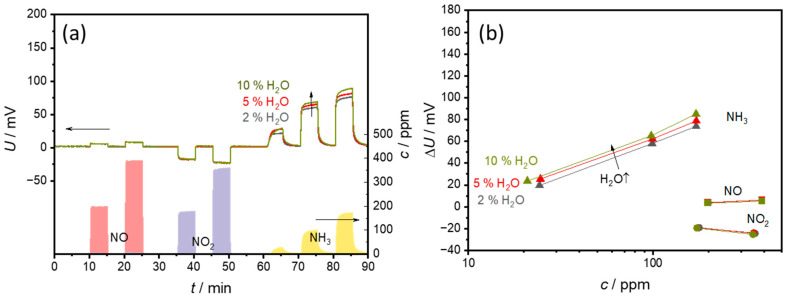
Effect of a water concentration of 2%, 5%, or 10% water in the base gas (20% O_2_ in N_2_) on (**a**) the sensor signal and (**b**) the sensor characteristic curves. The points correspond to the sensor voltages measured at each concentration level. The lines that connect these points serve only as a guide for the eye.

The examination of the influence of water vapor (2, 5, and 10% H_2_O) with 20% O_2_ in the base gas is illustrated in Figure 10. Figure 10a displays the measured voltages. The data show that water vapor influences the sensor behavior only slightly. The situation is similar for NO and NO_2_, where the measurements also yield almost identical signal curves with no detectable water vapor influence. Slight fluctuations in the sensor voltages only occur with NH_3_ and water vapor mixtures. For example, slightly higher voltages are measured with a higher water vapor content together with NH_3_.

The characteristic curve in Figure 10b shows that the water vapor content ∆*U* shifts to slightly higher voltages. The sensitivity, i.e., the slope of the characteristic curve, remains largely the same for all water vapor contents.

The stable basic signal indicates that no additional reactions occur at the electrodes due to the changing water vapor contents. Or the potential at both electrodes is changed to the same extent, so that no water-dependent voltage change can be measured. The only effects of water vapor occur together with NH_3_. Since water is present in reaction Equation (2), it is quite likely that it influences the reaction. However, it should inhibit rather than accelerate the reaction due to the reaction equilibrium, which means that lower ∆*U* should be measured. It is possible that the reaction is not inhibited to the same extent at both electrodes. As the VWT-covered electrode makes the largest contribution to the ammonia signal, the reaction should be inhibited more strongly at the uncovered electrode [25]. However, this theory could only be tested using a two-chamber setup with different gas atmospheres at the electrodes.

## 4. Conclusions

Ammonia sensors were manufactured and tested using a solid electrolyte layer of PAD, indicating its suitability for electrolyte application. In fact, the signals exceeded those of sensors with a screen-printed solid electrolyte. The sensors showed long-term stable signals over at least 70 h. The characteristic curve of the sensors depended strongly on the operating temperature. At lower temperatures, the sensor was more sensitive. The desired concentration range might, therefore, be adjusted via the sensor temperature. The cross-sensitivity to NO was very low. Compared to that, the NO_2_ cross-sensitivity was significantly higher, but with inverse polarity. Oxygen had a significant influence on the ammonia sensitivity of the sensor, so we surmise that the oxygen concentration must be known or measured during operation. H_2_O only slightly influenced the sensor behavior. The high suitability of PAD positions it as an interesting candidate for other solid electrolyte applications, especially for temperature-sensitive production processes.

## Data Availability

All relevant data presented in this article are stored according to institutional requirements and as such are not available online. However, all data used in this paper can be made available upon request to the authors.
